# Shear wave elastography can stratify rectal cancer response to short-course radiation therapy

**DOI:** 10.1038/s41598-023-43383-5

**Published:** 2023-09-26

**Authors:** Reem Mislati, Taylor P. Uccello, Zixi Lin, Katia T. Iliza, Kimani C. Toussaint, Scott A. Gerber, Marvin M. Doyley

**Affiliations:** 1 Department of Electrical and Computer Engineering, University of Rochester, Rochester, NY USA; 2 Department of Microbiology and Immunology, University of Rochester, Rochester, NY USA; 3 School of Engineering, Brown University, Providence, RI USA; 4 Department of Biomedical Engineering, University of Rochester, Rochester, NY USA; 5 Department of Surgery, University of Rochester, Rochester, NY USA

**Keywords:** Biomedical engineering, Diagnostic markers, Cancer imaging, Cancer microenvironment

## Abstract

Rectal cancer is a deadly disease typically treated using neoadjuvant chemoradiotherapy followed by total mesorectal excision surgery. To reduce the occurrence of mesorectal excision surgery for patients whose tumors regress from the neoadjuvant therapy alone, conventional imaging, such as computed tomography (CT) or magnetic resonance imaging (MRI), is used to assess tumor response to neoadjuvant therapy before surgery. In this work, we hypothesize that shear wave elastography offers valuable insights into tumor response to short-course radiation therapy (SCRT)—information that could help distinguish radiation-responsive from radiation-non-responsive tumors and shed light on changes in the tumor microenvironment that may affect radiation response. To test this hypothesis, we performed elastographic imaging on murine rectal tumors (n = 32) on days 6, 10, 12, 16, 18, 20, 23, and 25 post-tumor cell injection. The study revealed that radiation-responsive and non-radiation-responsive tumors had different mechanical properties. Specifically, radiation-non-responsive tumors showed significantly higher shear wave speed SWS (p < 0.01) than radiation-responsive tumors 11 days after SCRT. Furthermore, there was a significant difference in shear wave attenuation (SWA) (p < 0.01) in radiation-non-responsive tumors 16 days after SCRT compared to SWA measured just one day after SCRT. These results demonstrate the potential of shear wave elastography to provide valuable insights into tumor response to SCRT and aid in exploring the underlying biology that drives tumors' responses to radiation.

## Introduction

Colorectal cancer is a leading cause of cancer-related death in the United States^1^, and neoadjuvant chemoradiotherapy, followed by total mesorectal excision, is the standard of care for locally advanced rectal cancer^2^. Total mesorectal excision surgery can cause major side effects, such as urinary and sexual dysfunction, and may not be necessary for all patients. To minimize complications, clinicians use conventional imaging, such as computed tomography (CT) or magnetic resonance imaging (MRI), to assess the tumor response to neoadjuvant therapy^3–5^ before surgery. This strategy is called the “watchful waiting”^6,7^ approach. Patients undergoing neoadjuvant therapy are closely monitored to assess tumor response. Alternate imaging techniques can provide valuable insights into how tumors respond to therapy by showing changes in intrinsic tissue properties in response to changes in the colorectal tumor microenvironment. Researchers can use this information to gain insight into the biological response of tumors to treatment. It can also improve the monitoring of a broader range of therapies for rectal cancer response.

Functional imaging methods, such as dynamic contrast-enhanced MRI (DCE-MRI) and elastography, have been developed as alternate approaches to MRI or CT for evaluating tumor response to neoadjuvant therapy. DCE-MRI provides information about tumor biology and has been shown to correlate strongly with angiogenesis. However, quantitative methods using DCE-MRI^8^ have yielded conflicting results^9,10^. Elastography is a technique that measures changes in the mechanical properties of tissues in vivo*,* which can provide information regarding extracellular matrix (ECM) remodeling^11,12^, a key indicator of tumor progression. The shear modulus of cancerous rectal tumors is higher than that of healthy rectums, while the viscosity of cancerous rectal tumors is lower than healthy rectums as demonstrated by Deptula et al*.*^13^. Using endoscopic ultrasound elastography, Esaki et al*.*^14^ demonstrated that elastographic assessment (accuracy and sensitivity) of colorectal neoplasms was comparable to chromoendoscopy, which is the gold standard for diagnosing the degree of neoplasm invasion. Using magnetic resonance elastography to characterize the shear modulus of murine colorectal tumors, researchers observed that tumors treated with a vascular disrupting agent that promotes cancer cell necrosis decreased the shear modulus^15^. Although elastography improves the assessment of tumor response to neoadjuvant therapy, more research is needed to understand the relationship between elastographic parameters and rectal cancer treatment response and prognosis to improve the technique for clinical use.

This research tests the hypothesis that shear wave elastography (SWE) can assess the response of rectal cancer to short-course radiation therapy (SCRT). To corroborate this, SWE imaging was performed on murine rectal tumors undergoing SCRT; this murine model was designed to replicate the SCRT response rate observed in the clinical setting^16^. Healthy rectums were used as positive controls, untreated tumors were used as negative controls, and radiation-responsive and radiation-non-responsive tumors were evaluated on days 6, 10, 12, 16, 20, 23, and 25 post-injections. Shear wave speed (SWS) and shear wave attenuation (SWA) measurements were correlated with changes in tumor cross-sectional area and with other methods such as quantitative histological analysis, second harmonic generation imaging, genetic analysis, and bioluminescence imaging to understand better how biological changes within the tumors during therapy impact SWE. Shear wave speed is the velocity at which a shear wave travels through tissue and is linked to its shear modulus and density. In contrast, shear wave attenuation is the loss of energy incurred when a shear wave travels through tissue and is linked to viscosity.

## Methods

This section describes the tumor line, murine model, radiation treatment, imaging modalities (SWE, bioluminescence imaging, and second harmonic generation imaging), and histological and statistical analysis employed in this study. All experiments were performed using protocols approved by the University of Rochester Committee on Animal Resources (UCAR), in compliance with University of Rochester Medical Center guidelines, and were consistent with ARRIVE (Animal Research: Reporting of In Vivo Experiments) guidelines.

### Tumor model

This study was performed on 6-to-8-week-old female C57BL/6J mice (Jackson Laboratory, Bar Harbor, ME, USA). Colon 38-luciferase-expressing tumor cells (2.5 × 10^4^/5 μL) were mixed in a 1:1 ratio of Evans blue to Matrigel matrix (BD Bioscience, Mississauga, Canada). The tumor cells were injected orthotopically into the rectal wall as described by Uccello et al.^16^. Table 1 includes the number of tumors in each group, and the subsets of tumors excised on days 20 and 25 post-injection.

**Table 1:** Sample number of untreated tumors, healthy irradiated rectum, radiation-non-responsive and radiation-responsive tumors.

Group	Excised on day 20 post injection (day 11 post-SCRT)	Excised on day 25 post injection (day 16 post-SCRT)	Total
Untreated (control) tumors	5	10	15
Healthy irradiated rectum	5	0	5
Radiation-non-responsive tumors	4	13	17
Radiation-responsive tumors	6	4	10

### Short-course radiation therapy

A total of 32 mice (27 tumor-bearing mice and five healthy mice (positive controls)) received SCRT. Fifteen additional tumor-bearing mice (negative controls) did not receive SCRT. Before treatment, two 4 mm titanium fiducial markers (Horizon, Teleflex, Morrisville, NC, USA) were placed on opposing sides of the rectal tumor to aid in CT-guided SCRT targeting as described in Uccello et al*.*^16^. A small animal radiation research platform (SARRP, Xstrahl Inc., Suwanee, GA, USA) with a 5 mm collimator was used to irradiate mice in the treated group with 5 Gy (Gy) from day 9 to 13 post-tumor cell injection.

### Bioluminescence imaging

We used an IVIS^®^ imaging system (PerkinElmer Inc., Waltham, Massachusetts) to monitor tumor growth by assessing bioluminescence (BL). Mice were anesthetized with vaporized isoflurane, injected with D-Luciferin (75 mg/kg, Invitrogen), and 12 consecutive images were taken at 2 min intervals. The maximum BL over the 12 images within the tumor region of interest was recorded. Radiance was measured on the same day SWE was performed except for radiation week. In this study, tumors with an average radiance above 10^6^ on day 20 were classified as radiation-non-responsive. In contrast, those with a mean radiance below this threshold were classified as radiation-responsive. To ensure we accurately classified the tumors as responders or non-responders based on BLI, we excised all tumors on day 20 and weighed them. Tumors weighing less than 0.005 g were considered responsive, while heavier tumors were considered non-responsive, as demonstrated in^17^.

### Shear wave elastography imaging

We used plane-wave single tracking location shear wave elastography (pSTL-SWE) on a commercially available ultrasound scanner (Vantage 256, Verasonics Inc., Kirkland, WA, USA) equipped with an 11-5v linear transducer array (Vantage 256, Verasonics Inc., Kirkland, WA, USA) to perform SWE^18^. Tumors were first located using a metal rod (1.35 mm diameter by 20 mm length) before acquiring three cross-sectional (~ 2 mm increments) SWE images. In addition, we used the bladder and the metal clips on both ends of the tumor to localize the tumor, as illustrated in (Supplementary Fig. 1). The tumor segmentation was performed in the ultrasound images. The cross-sectional area was computed for three acquired cross-sections, and the mean cross-sectional area was used as a surrogate of tumor volume. SWS and SWA values were computed using the time-of-flight^18^ and frequency shift^19^ methods. pSTL-SWE imaging was performed at three different times: before injecting tumor cells (day 0), during treatments (days 10 and 12 post-tumor cell injection), and post-SCRT (days 7, 9, 11, 14, and 16). We used ultrasound images to guide us as we manually segmented all tumor cross-sections. We calculated the mean SWS and SWA for each time point across all the segmented regions.

### Histological analysis

A group of tumors were excised 11 days after SCRT, classified as radiation-responsive or radiation-non-responsive, and fixed in 10% neutral buffered formalin (Azer Scientific, Morgantown, PA, USA). Excised tumors were embedded in paraffin before sectioning in 5 μm increments. Each tumor slice was stained with trichome (New Comer, Middleton, WI, USA) and digitized. We used the ImageScope Software package (Leica Biosystems, Nussloch, Germany) to quantify collagen density from the digitized histological images.

### Second harmonic generation microscopy

We used a multiphoton microscope (Olympus FV-1000-MPE, Olympus, Tokyo, Japan) equipped with a tunable laser (Mai Tai HP, Spectra-Physics, Santa Clara, CA, USA) to assess whether the orientation of collagen in radiation-responsive and radiation-non-responsive tumors was different. We acquired optical images from excised tumors on day 11 post-SCRT. We used a laser with an excitation wavelength of 800 nm and a 405/40 filter cube to capture second harmonic generation (SHG) signals. We used a 25 × (numerical aperture 1.05) objective lens to acquire volumetric images (254 × 254 × 38 µm) and to estimate the orientation of the SHG image stack as described in^20,21^. We calculated the average and standard deviations of the in-plane and out-of-plane orientations (i.e., the spherical variance, spherical theta (θ), and spherical phi (φ)) to determine differences in collagen orientation between the two groups of tumors.

### Gene expression analysis

Gene set enrichment analysis (GSEA) was performed on radiation-responsive and radiation-non-responsive tumors^22,23^. Excised tumors were incubated in 30% collagenase (Sigma-Aldrich, St. Louis, MO, USA) for 30 min at 37 °C, and the homogenates were separated with 40 μm diameter filters. After the cells were resuspended in PAB (1L PBS, 1 g sodium azide, 10 g BSA), we stained approximately 2 × 10^6^ to 4 × 10^6^ cells for surface antigens (30 min, 4 °C). A FACSAria II cell sorter (BD Biosciences, Haryana, India) equipped with a 50 μm nozzle was used to sort CD45−, CD31−, and C38.GFP−, Podoplanin+, PDGFRa+, and Ly6C+ cancer associated fibroblasts (CAFs). The sorted CAFs were lysed in Buffer RLT (containing β-mercaptoethanol) (QIAGEN, Germantown, MD, USA) and homogenized using QIAShredder (QIAGEN, Germantown, MD, USA) spin columns. The University of Rochester Genomics Research center performed RNA sequencing on the purified RNA samples. The RNA quality was evaluated using a bioanalyzer (Agilent, Santa Clara, CA, USA), and the tissue samples had RNA integrity values greater than 5.

### Statistical analysis

All statistical analysis was performed using Prism 9 software (GraphPad, La Jolla, CA). We used a Mann–Whitney test to analyze the statistical significance between radiation-responsive and non-responsive tumors. Additionally, we used a Kruskal–Wallis test to compare the collagen density of untreated, radiation-non-responsive, and radiation-responsive tumors. To further analyze changes in SWS, SWA, and cross-sectional area compared to day 1 post-therapy, we also conducted a Dunn's multiple comparison test.

## Results

### Characterization of SWS, SWA, and radiance in tumor in response to SCRT

**Figure 1:**
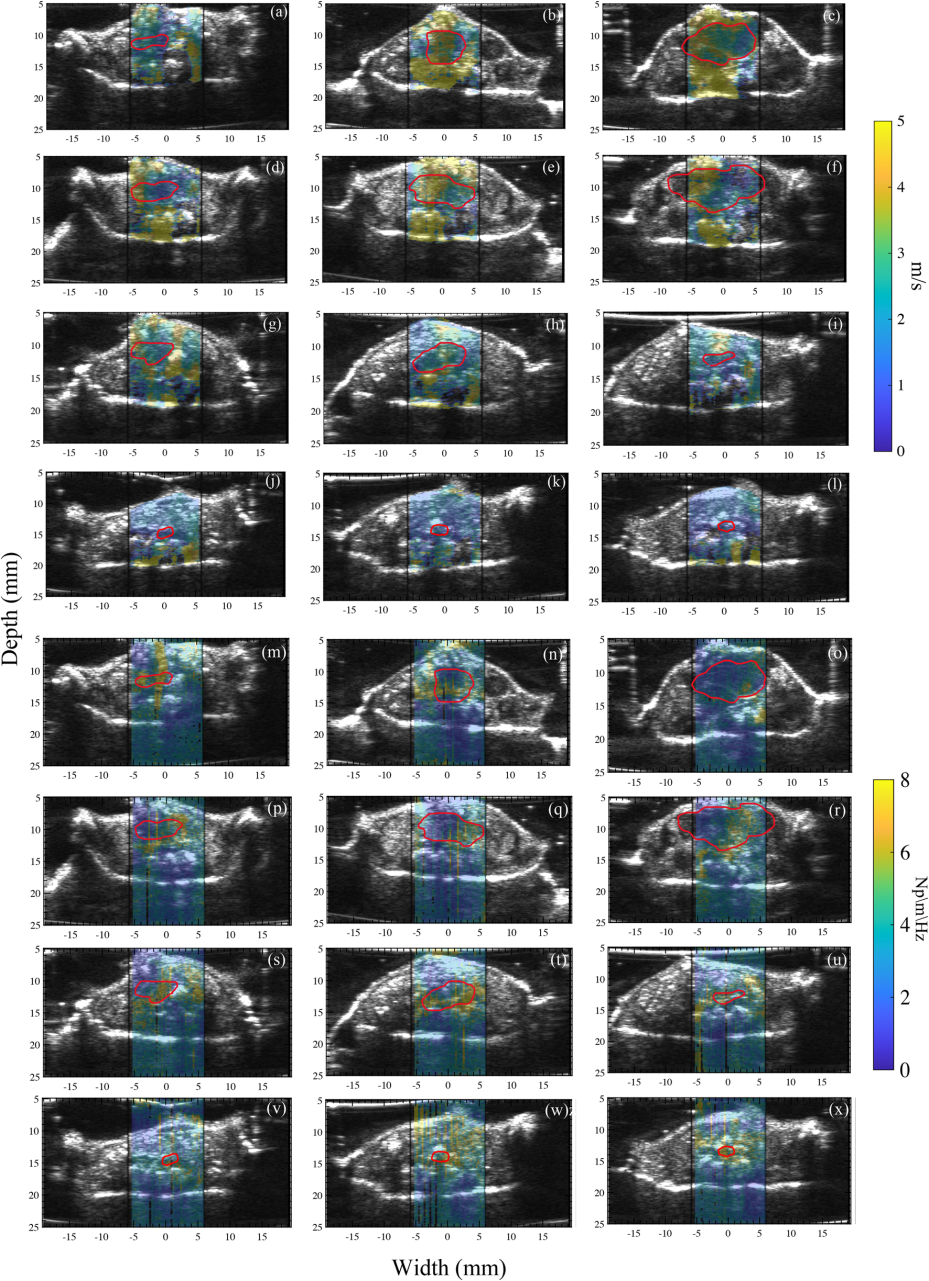
Sonograms displaying the shear wave speed (SWS, **a**–**l**) and shear wave attenuation (SWA, **m**–**x**) of representative samples where tumors are outlined in red. The samples include untreated tumors (**a**–**c**), tumors that were radiation-non-responsive to short-course radiotherapy (SCRT) (**d**–**f**), tumors that were responsive to SCRT (**g**–**i**), and healthy rectum tissue (**j**–**l**). The columns in the figure represent the SWS or SWA measurements taken on days 12, 16, and 20 post-injection (3, 7, and 11 post-SCRT). The sonograms with SWA are also shown for untreated (**m**–**o**), radiation-non-responsive tumors (**p**–**r**), radiation-responsive tumors (**s**–**u**), and healthy rectum (**v**–**x**). This figure provides a clear understanding of how SWS and SWA changes in different tissue types over time compared to healthy rectum tissue.

**Figure 2:**
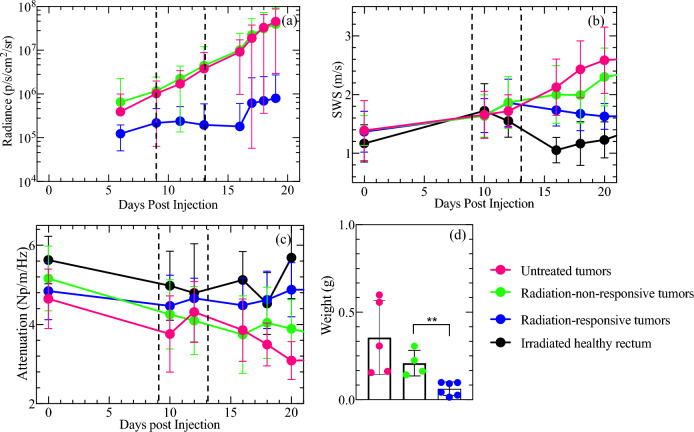
Measured tissue properties (radiance, SWS, SWA) of untreated tumors (pink), radiation-non-responsive tumors (green), radiation-responsive tumors (blue), and health rectum (black). Subplot (**a**) shows the radiance measured from day six post-tumor cell injection. Subplots (**b**) and (**c**) show the SWS and SWA measured over 20 days post-tumor cell injection, respectively. The vertical dotted lines indicate two time periods when measurements were made: during treatment (between the lines) and after therapy. Subplot (**d**) shows the weight of excised tumors 20 days post-tumor cell injections. The data reveals that SWS and radiance of untreated and radiation-non-responsive tumors increased with time; SWS and radiance of radiation-responsive tumors were lower than untreated and radiation-non-responsive tumors. The SWS of the normal rectum increased rapidly, decreased from days 10 to 16, and then increased steadily after day 16. SWA of untreated and radiation-non-responsive tumors was noticeably lower than those of healthy rectum and radiation-responsive tumors. Additionally, radiation-responsive tumors weighed significantly less than radiation-non-responsive tumors, as indicated by the p-value in the figure (**p < 0.01).

**Figure 3:**
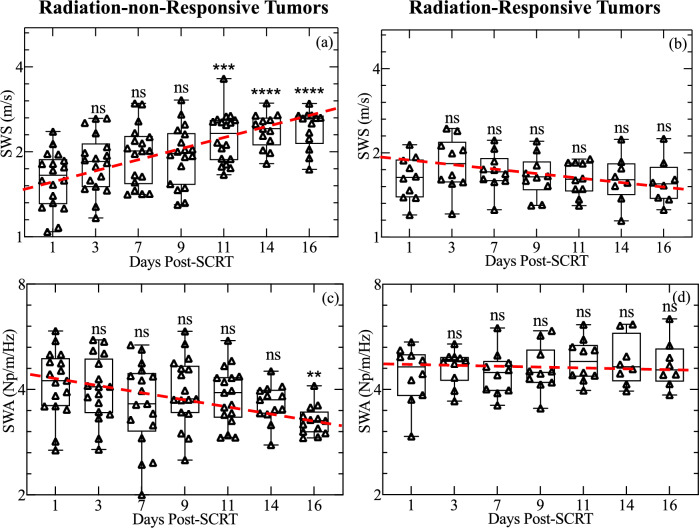
Changes in SWS and SWA for radiation-non-responsive and radiation-responsive tumors measured on days 1 to 16 post-SCRT. The boxplots in (**a**) and (**b**) show the changes in SWS measured up to 16 days post-SCRT for the subset of radiation-non-responsive and radiation-responsive tumors, respectively. Similarly, the boxplots in (**c**) and (**d**) show the change in SWA for the same subset of tumors for radiation-non-responsive and radiation-responsive tumors, respectively. The dashed red line is a visual guide of the trend observed over time. The significance was computed using a Kruskal–Wallis test followed by a Dunn’s multiple comparison test where the day 1 post-SCRT was set as control. The data reveals that SWS and SWA of radiation-non-responsive tumors changed significantly after SCRT, but there was no significant change in the properties of radiation-responsive tumors after SCRT. The significance of the differences is indicated by p-values and calculated relative to day 1 post-SCRT, with ** indicating p < 0.01, *** indicating p < 0.001, **** indicating p < 0.0001, and ns indicating p > 0.05.

Elastographic images showed that untreated (Fig. 1a–c) and radiation-non-responsive tumors (Fig. 1d–f) increased in SWS over time while radiation-responsive and healthy rectal tissue had decreased stiffness (Fig. 1g–l). Quantitative analysis confirmed this observation, with the SWS of radiation-non-responsive tumors increasing steadily (Fig. 2b) and that of radiation-responsive tumors decreasing (Fig. 2b). The radiance of untreated and radiation-non-responsive tumors also increased linearly over time (Fig. 2a), indicating increased tumor burden. Radiation-responsive tumors had consistently lower radiance and relatively constant tumor burden over time. We also observed an inverse relationship between the SWA of radiation-non-responsive tumors and time (Fig. 2c), with the SWA of radiation-non-responsive (Fig. 1p–r) and untreated (Fig. 1m–o) tumors noticeably lower than those of radiation-responsive tumors and healthy rectum (Figs. 1s–x and 2b). Statistical analysis revealed significant differences in the SWS of radiation-non-responsive tumors on days 11, 14, and 16 post-SCRT compared to day 1 (p-value of 0.0010, < 0.0001, and < 0.0001, respectively). We observed a significant difference in SWA only on day 16 post-SCRT compared to day 1 (p of 0.0031) (Fig. 3a,c). No significant difference was observed in either SWS or SWA of radiation-responsive tumors on days 11, 14, and 16 post-SCRT compared to day 1 (Fig. 3b,d).

### Changes in tumor size in response to SCRT

**Figure 4:**
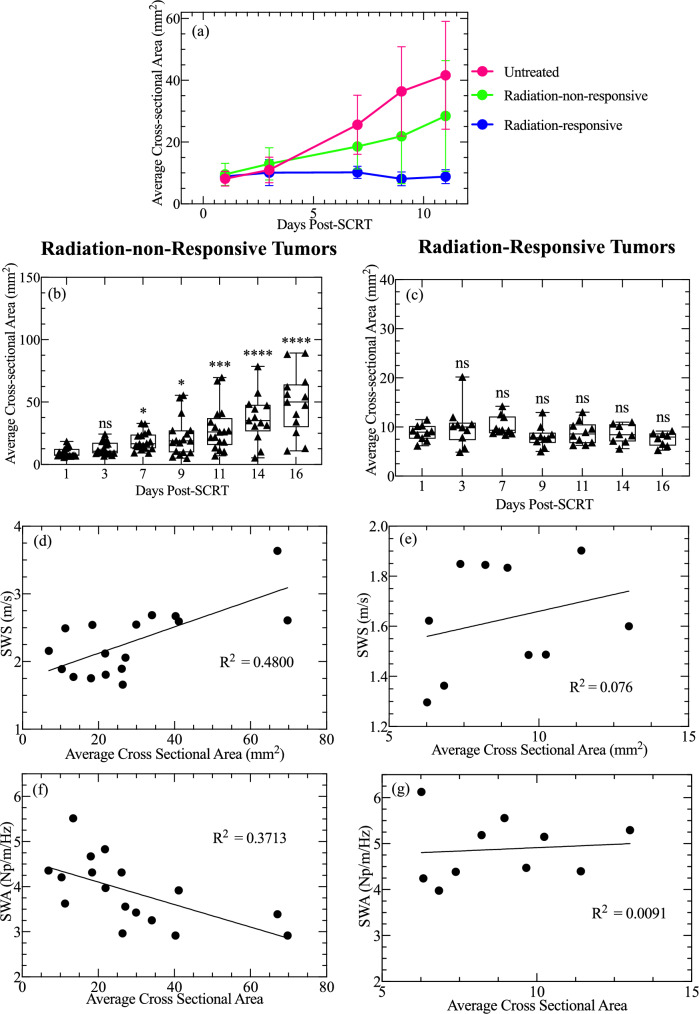
Tumor area of untreated, radiation-non-responsive, and radiation-responsive tumors as a function of days post-SCRT. Subplot (**a**) shows the average cross-sectional area computed for the untreated (pink), radiation-non-responsive (green), and radiation-responsive tumors (blue). The untreated and radiation-non-responsive tumors exhibit an increase in area over time. Subplot (**b**) shows the cross-sectional area of the radiation-non-responsive tumors as a function of days post-SCRT. The significance is computed using a Kruskal–Wallis test with multiple comparisons. Subplot (**c**) shows the cross-sectional area of radiation-responsive tumors. There is no significant change in the cross-sectional area for the radiation-responsive tumors. However, there is a significant change for radiation-non-responsive tumors starting day 7 post-SCRT. The significance of the difference is indicated by p-values, with **** indicating p < 0.0001, ** indicating p < 0.005 and ns indicating p > 0.05. Subplots (**d**) and (**e**) show the correlation between SWS and the cross-sectional area on day 11 post-SCRT for radiation-non-responsive and radiation-responsive, respectively. Subplots (**f**) and (**g**) show the correlation SWA and the cross-sectional area of radiation-non-responsive and radiation-responsive tumors, respectively. This figure highlights that the tumors cross-sectional area changes at an earlier time point than SWS and SWA. There is a weak correlation between size and SWS for both groups.

Figure 4 shows the tumor area as a function of days post-SCRT. After day 3, the untreated and radiation-non-responsive group displayed a drastic increase in area while the radiation-responsive size remained consistent (Fig. 4a). The radiation-non-responsive tumors showed no significant change in size from day 1 to 3 post-SCRT. However, there is a significant increase in size on day 7, 9, 11, 14, and 16 (p-values 0.0203, 0.0200, 0.0002, < 0.0001, and < 0.0001, respectively) (Fig. 4b). The size of radiation-responsive tumors did not significantly change compared to day 1 post-SCRT as depicted in Fig. 4c. Figure 4d,e shows the results of linear regression analysis between SWS and tumor size 11 days post-SCRT. The analysis revealed that R^2^ was 0.480 and 0.076 for radiation-non-responsive and radiation-responsive tumors, respectively, indicating a weak correlation between size and SWS. Additionally, there was a weak correlation between SWA and size with R^2^ values of 0.371 and 0.009 for radiation-non-responsive and radiation-responsive tumors, respectively.

### Histological analysis of collagen density and alignment of radiation-responsive and radiation-non-responsive tumors

**Figure 5:**
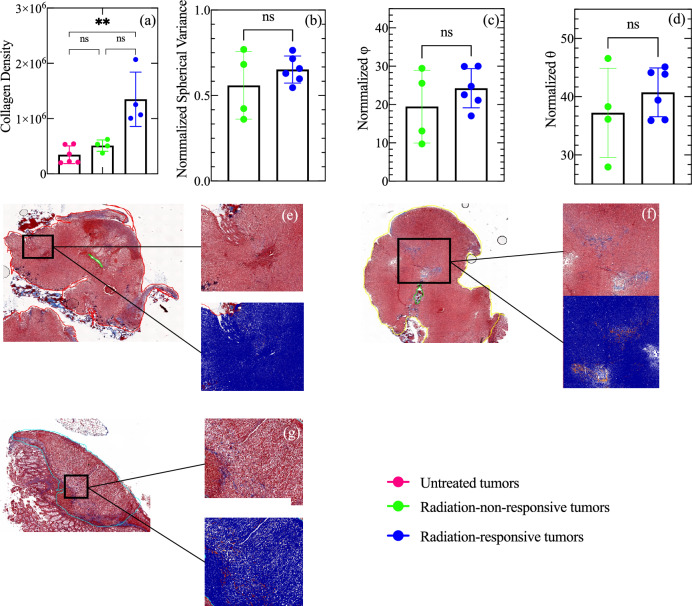
Histological assessment of untreated (solid pink circles), radiation-non-responsive (solid green circles), and radiation-responsive tumors (solid blue circles). (**a**) Collagen density of the three tumor groups. (**b**–**d**) Box plots of the parameters denoting collage orientation (normalized variance, *φ*, *θ*, respectively). (**e**–**g**) Digitized Masson’s Trichrome of untreated, radiation-non-responsive, and radiation-responsive tumors, respectively, with zoom regions of areas of high collagen density (collagen illustrated by blue staining). The results show no statistically significant difference in either collagen density or collagen orientation of radiation-non-responsive and radiation-responsive tumors. The significance of the differences is indicated by p-values, with ** indicating p < 0.01 and ns indicating p > 0.05. The collagen density of untreated tumors was too low to guarantee meaningful results with SHG, so Fig. 6b–g only reports the results of treated tumors.

Histological samples were analyzed to determine if the radiation regimen employed in this study changed collagen density or collagen alignment. Kruskal–Wallis’s test revealed significant differences between the three groups of tumors (p-value = 0.0019). However, Dunn’s multiple comparison tests showed that only the collagen densities of the untreated and radiation-responsive tumors were significantly different (p-value = 0.008) (Fig. 5a). There was no significant difference in collagen density between untreated vs. radiation-non-responsive tumors or radiation-non-responsive vs. radiation-responsive tumors (both with p-values > 0.01). We observed no significant difference in the orientation of collagen fibers of radiation-responsive and radiation-non-responsive tumors (Fig. 5b–d). We also observed low collagen density in all tumors, particularly the untreated groups (Fig. 5e–g).

### Gene set enrichment analysis of the extracellular matrix

To investigate the genetic differences between radiation-responsive and radiation-non-responsive tumors, we compared the gene expression data of both groups with respect to radiation-responsive tumors using thresholds of 0.05 and 1 for adjusted p-value and the log two-fold change, respectively. We identified 50 genes over-expressed in the radiation-responsive group and 274 genes over-expressed in the radiation-non-responsive group.

**Figure 6:**
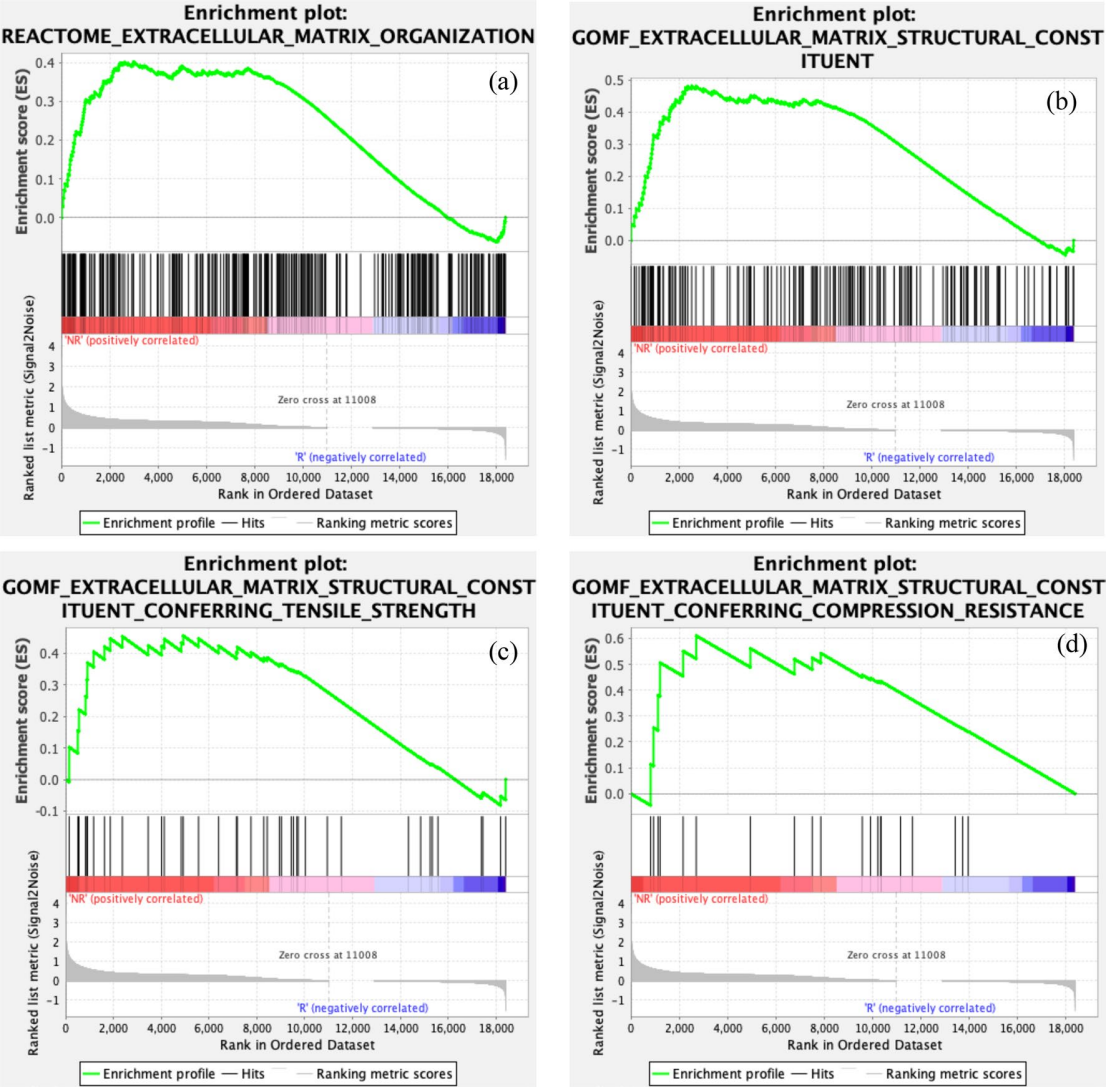
Results of genetic analysis of tissue samples from radiation-non-responsive, and radiation-responsive tumors. The enrichment plots for gene ontology pathways related to the ECM in the radiation-non-responsive group are presented in subplots (**a**–**d**). The data suggest that radiation-responsive tumors do not show significant gene ontologies, while radiation-non-responsive tumors exhibit essential gene ontology items related to ECM. The ECM of the radiation-non-responsive tumors is more resistant to compressive force than those of radiation-responsive tumors.

Using GSEA and comparing radiation-non-responsive genes to radiation-responsive genes, we observed that radiation-responsive tumors did not express critical gene ontology pathways related to ECM. In contrast, radiation-non-responsive tumors expressed considerable gene ontology pathways related to ECM (Fig. 6a–d). Among these pathways, ECM organization and structural changes were observed in the radiation-non-responsive group (Fig. 6a–d). The latter enrichment plots show that the ECM of radiation-non-responsive tumors is more resistant to compressive force than those of radiation-responsive ones.

We also observed a similar expression of collagen types in both radiation-non-responsive and radiation-responsive tumors (Supplemental Fig. 2a), with no significant difference in collagen I and III expressions between the two groups (Supplemental Fig. 2b). When comparing radiation-non-responsive to untreated tumors, we found radiation-non-responsive tumors over-expressed collagen I, IV, V (a1, a3), and VI (Supplemental Fig. 2c). Similarly, when comparing radiation-responsive tumors to untreated tumors, we observed that the radiation-responsive tumors over-expressed collagen I, IV, VI(a), and XII (Supplemental Fig. 2c). The collagen I (a1) gene was upregulated in the irradiated group (Supplemental Fig. 2c). However, collagen III, abundant in colorectal tumors^24^, did not show a significant difference in expression between the untreated and irradiated groups.

## Discussion

This paper hypothesizes that rectal cancer tumors responsive to SCRT can be differentiated from non-responsive tumors based on their mechanical properties, specifically shear wave speed (SWS) and shear wave attenuation coefficient (SWA). To test this hypothesis, we used SWE to measure the SWS and SWA of murine rectal tumors treated with SCRT. The main findings of this study are as follows:

- Radiation-non-responsive and untreated tumors had significantly higher SWS than radiation-responsive tumors on day 11 post SCRT (Figs. 2, 3).
- Radiation-responsive tumors' SWA was higher than radiation-non-responsive tumors from 16 days post-SCRT (Figs. 2, 3).
- The collagen density of tumors treated with radiation was higher than untreated tumors. However, the collagen density of radiation-responsive and radiation-non-responsive tumors was similar, as shown in Fig. 5a.
- Radiation-non-responsive tumors had a significant increase in size starting day 7 post-SCRT which is earlier than SWS. However, there is no correlation between size and SWS (Fig. 4d).

Solid stress changes during tumor progression^25^, and is the most likely factor responsible for the observed difference in SWS of radiation-responsive and radiation-non-responsive tumors. Radiation-responsive tumors regress steadily to a stable state, unlike their radiation-non-responsive counterparts that continue to grow, which is reflected by differences in the weight of the excised radiation-responsive and radiation-non-responsive tumors (Fig. 2d). As cancer grows, it exerts mechanical stress on the extracellular matrix and the stromal cells^26^, which increases solid stress, resulting in higher shear modulus^27^ and SWS. In contrast, as the tumor regresses, solid stress and SWS decrease. Figure 3c,d demonstrates that the SWA of radiation-responsive tumors is generally higher than untreated tumors and their radiation-non-responsive counterpart. To understand this, it is important to know that the viscosity of the fluid component of tumors influences SWA. As the tumor grows, new vascular networks are formed to provide cancer cells with nutrients and oxygen and to remove waste products. However, in radiation-non-responsive tumors, the higher solid stress can compress vessels, which reduces blood flow. The reduction in the blood flow^28^ creates a hypoxic and acidic tumor microenvironment, decreasing the efficacy of radiation therapy. Since SWA is linked to viscosity, reduction in viscosity leads to lower SWA in radiation-non-responsive tumors^29^. Researchers have shown that radiation reduces ECM stiffening^30^, and can induce epithelial damage in the rectum^31^. We attributed the observed decrease in SWS in healthy rectums exposed to radiation to the combined effects of epithelial damage and reduced ECM stiffening (see Fig. 2b). We plan to perform more detailed studies to confirm this hypothesis.

Collagen is a protein that is present in the ECM^32^, and plays a vital role in tumors progression^24,33^ and the mechanical properties of normal and diseased tissues^34^. To assess if collagen was responsible for the observed differences in the sensitivity to radiation, we measured the collagen density of radiation-responsive and radiation-non-responsive, and untreated tumors (control). Figure 5a demonstrated that the collagen density of radiation-treated tumors was noticeably higher than that of untreated tumors (p = 0.0075), likely due to an increase in collagen deposition in response to radiation therapy, consistent with previously reported research. Using atomic force microscopy, Kotova et al*.*^35^ observed significant fibrosis (increased collagen deposition) in the ECM of rectal tissue treated with radiation therapy. This suggests that the observed difference in collagen density between untreated and radiation-treated tumors (Fig. 5a) may represent the onset of fibrosis, a condition characterized by excessive collagen deposition in tissue. However, the collagen density of radiation-responsive and radiation-non-responsive tumors was similar, suggesting that other factors in the tumor microenvironment were responsible for the observed differences in mechanical properties. It is possible that differences in the tumor volume of radiation-responsive and radiation-non-responsive tumors could be a factor, but Fig. 4d–g shows a weak correlation between mechanical properties (SWS and SWA) and tumor size (cross-sectional area) for radiation-non-responsive tumors. There was no correlation between mechanical properties (SWS and SWA) and tumor size for radiation-responsive tumors. This implies that tumor size was not the primary mechanism responsible for the observed differences in mechanical properties. Other factors, such as differences in immune response, angiogenesis, or hypoxia between radiation-responsive and radiation-non-responsive tumors, could be responsible. This study did not assess how these factors impact mechanical properties, but we plan to evaluate them in future studies and communicate our findings in future communications in this journal.

The orientation of collagen fibers also affects the mechanical properties, and researchers have shown that when collagen fibers are aligned, tissue stiffness is lower^36^. Tumors treated with radiation have an anisotropic collagen fiber distribution, while untreated tumors have an isotropic distribution^35^. In this study, we could not measure collagen alignment in untreated tumors due to low collagen density but found that the collagen alignment of radiation-responsive and radiation-non-responsive tumors was similar (Fig. 5b–d), suggesting that the observed difference in SWS is likely due to some other factor, such as differences in solid stress.

Figure 3 demonstrates that rectal tumors that respond to radiation have significantly lower SWS 11 days after SCRT compared to those that do not respond. However, it is essential to determine if the number of days post-SCRT can be reduced to stratify the two groups of tumors. One possibility is to combine information measured with contrast-enhanced ultrasound imaging with elastographic imaging. The figure also demonstrates that two groups of tumors can be stratified based on SWA, likely due to differences in solid stress. However, this may differ for therapies that reduce tumor size more slowly. Consequently, we plan to conduct further studies to compare the efficacy of SWS and SWA to monitor the rectal tumor response to cytostatic drugs such as sunitinib and bevacizumab.

This study has four main limitations that should be considered. Firstly, we did not directly measure the interstitial tissue pressure during the study. This is important since it has been shown that an increase in solid stress incurred during tumor progression increases the interstitial tissue pressure^27,37^. Additionally, the tumors tend to be small at early points, making it difficult to measure SWS and SWA accurately. Secondly, using only 2D measurements to evaluate the response of tumors may lead to inaccuracies when determining the efficacy of SCRT. To address this in future research, we aim to create shear wave elastography based on 3D ultrasound imaging or utilize magnetic resonance elastography. In clinical settings, MR and CT scans are considered the most dependable methods of assessing tumor response to therapy. Therefore, we also plan to compare the measurements of tumor response obtained from a small animal MRI system with those acquired using SWE in future studies. Thirdly, we did not quantify the collapse of vasculature in radiation-non-responsive and untreated tumors; therefore, our explanation for the observed difference in SWA is speculative. In future studies, we plan to address this limitation by using fluorescence lectin to quantify the tumor vessels in radiation-responsive and radiation-non-responsive tumors to understand better the effects of solid stress on vessels and SWA^38,39^. Finally, only one tumor model was employed. The murine model employed in this study offers significant advantages in studying rectal tumors, as it produces a treatment response rate similar to what's observed in clinical settings and allows us to conduct studies using clinical doses. One of our long-term goals is to understand why some tumors in this model respond to radiation therapy while others don't. The results of our ongoing research suggest that the induction of type I interferon after SCRT is one of the factors responsible for the varying tumor responses^16^.

## Conclusion

In this research, we hypothesized that shear wave elastography in determining the response of rectal; tumors to short-course radiation therapy (SCRT). The study tested this hypothesis using SWE to measure the mechanical properties (SWS and SWA) of murine rectal cancer models before and after SCRT. The results showed that tumors that responded to radiation had lower SWS and higher SWA than radiation-non-responsive tumors. Additionally, there was no significant difference in collagen density or collagen orientation of radiation-non-responsive and radiation-responsive tumors. RNA sequencing of the CAFs revealed a heightened gene signature associated with ECM remodeling in radiation-non-responsive tumors compared to radiation-responsive tumors.

### Supplementary Information

Supplementary Figures.

## Data Availability

The datasets generated during the current study are not publicly available but are available from the corresponding author upon reasonable request.
