# Supplementary material for: Shear wave elastography can stratify rectal cancer response to short-course radiation therapy
Source: Sci Rep. 2023 Sep 26;13:16149. doi: 10.1038/s41598-023-43383-5 (PMC10522682; doi:10.1038/s41598-023-43383-5)
Supplement: Supplementary file 1 — Supplementary Figures. [file 41598_2023_43383_MOESM1_ESM.docx]

**Supplemental Figure 1**: Illustrating how tumors were located *in vivo*. Ultrasound images are acquired, starting from the bladder, and moving upwards. Once the bladder was found, (a) the metal clip can be seen as a bright object, with reflections (b). The tumor was found next to the metal clip, as shown in subplot (c). The tumor segmentation was segmented manually based on the ultrasound image.

**Supplemental Figure 2**: Results of genetic analysis for collagen types of tissue samples from untreated, radiation-non-responsive, and radiation-responsive tumors. Subplot (a) displays the normalized count of collagen genes in radiation-non-responsive (green) and radiation-responsive (blue) tumors, and subplot (b) shows the normalized count of collagen type I (a1), I (a2), and III in radiation-non-responsive and radiation-responsive tumors, indicating no significant difference in collagen (I and II) in the two groups of tumors. Subplot (c) shows the log 2-fold change for the collagen genes that were differentially expressed in radiation-non-responsive vs. untreated tumors and radiation-responsive vs. untreated tumors. Boxes with white background and a cross indicate that the specified gene was to not significantly expressed compared to the untreated group. This figure highlights the genes involved in collagen synthesis, how they differ in expression between irradiated and untreated tumors, and a comparison between radiation-responsive and radiation-non-responsive tumors.
